# Book Review: A Teacher’s Guide to Adapted Physical Education: Including Students with Disabilities in Sports and Recreation, 4th Edition

**DOI:** 10.3389/fpubh.2016.00197

**Published:** 2016-09-14

**Authors:** Jennifer J. Taylor

**Affiliations:** ^1^Health and Exercise Science, Western Oregon University, Monmouth, OR, USA

**Keywords:** adapted physical education, book review, teacher’s guide, sport and recreation, inclusion

The fourth edition of this book is organized into four sections. Section I: Foundations (Chapters  1–3) provides an in-depth description of physical education, inclusion, and taking a team approach to inclusion in physical education. Section II: Inclusive practices and Planning (Chapters 4–7) focus on planning and assessment along with instructional, curricular, game, and sport modifications. Section III: Understanding Specific Needs (Chapters 8–16) provides information related to understanding specific needs including intellectual, learning, and sensory disabilities as well as emotional disturbance. Section IV: Supporting Across Contexts (Chapters 17–21) focus on social acceptance, making physical education safe, behavior management, inclusion in community-based recreation, and multicultural education and issues of diversity. At the end of the book, there are comprehensive reference and index sections.

The fourth addition of this book has changed a number of areas from the previous third edition. First, this edition includes downloadable materials, which includes customizable PowerPoints for individuals who will be teaching a course using this book. Instructions on how to retrieve the material are also provided. Second, there is a notable difference in the number of collaborators for different chapters of the book. Block identifies in his acknowledgments how “thrilled he was to get some of the leaders in physical education for students with specific disabilities” to contribute to the new chapters. Third and perhaps the most notable change is the addition of nine chapters focusing on understanding the needs for specific disabilities (section III). Finally, block has changed some of the organization of the chapters. Most notably, (a) the book is now organized into four themed sections, (b) chapters 4 and 5 from the third edition (Planning for inclusion in physical education, assessment to facilitate successful inclusion) have been combined to create chapter 4 in the fourth edition (Program Planning and Assessment), (c) the aquatics chapter from the third edition has not been included in the newest edition, and (d) the new chapters on Understanding specific needs (chapters 9–16) have been inserted after the game and sport modifications chapter moving chapters on facilitating social acceptance, making inclusive physical education safe, positive behavior supports, community-based recreation, and multicultural education and diversity issues to comprise the last section of the book (Supporting Across Context). The organization of this book has an intuitive flow progressing from basic to more complex content regarding adapted physical education.

## Section I: Foundations (Chapters 1–3)

This section does an excellent job of describing quality physical education along with developmentally appropriate programing and curricular models. This section spends ample time on answering the question “what is inclusion?” and includes current research on inclusion in physical education as well as strategies for supporting inclusion in physical education. This section also spends an entire chapter on using a team approach to inclusion, discussing who is a part of the collaborative team (Physical Education Integration Team), and offers practical strategies for productive communication and managing conflict.

## Section II: Inclusive Practices and Planning (Chapters 4–7)

This section is filled with concrete ways to plan and modify curriculum, instruction, assessments, and games and sport. I especially liked the chapter on instructional modifications. This chapter (chapter 5) identifies different models related to modifications, selecting appropriate modifications, accommodations related to class organization, how information is presented, and providing structure and routine. This chapter also includes a well-designed sample peer tutoring training manual and training evaluation in the appendix.

## Section III: Understanding Specific Needs (Chapters 8–16)

Section III focuses on understanding specific disabilities (Intellectual Disabilities, Learning Disabilities, ADD/ADHD, Autism Spectrum Disorder, Emotional Disturbance, Deafness or Hard of Hearing, Visual Impairments and Deafblindness, Physical Disabilities, and Other Health Impairments). Each chapter describing the causes, incidence, and characteristics of various disabilities. Each chapter also provides instructional strategies, and modifications to help include students with these disabilities in physical education. Each chapter has some slight variation based on the specific needs for the disability that is being discussed. For example, chapter 11 (Autism Spectrum Disorder) spends time introducing behavior management strategies where as chapter 15 (Physical Disabilities) spends time discussing secondary health conditions, such as pressure sores and contractures. This section is very informative and well put together. At the end of each chapter is a list of current resources (more information on sport, camps, and support).

## Section IV: Supporting Across Contexts (Chapters 17–21)

This section has various topics including facilitating social acceptance, making inclusive physical education safe, positive behavior support, such as students with disabilities in recreation, and multicultural education and diversity issues. This section really rounds out this text book. It covers many of the topics that some may feel were not addressed in other areas and again provides current information and resources. The chapter on multicultural education and diversity issues explores issues related to disability and diversity like ableism, and individuals with disabilities as a minority group. The chapter also spends time discussing awareness of individuals with disabilities coming from a culturally diverse background and understanding the views of individuals with a disability and how they are represented in the media.

Overall, this book is an excellent choice for any adapted physical activity course that is preparing educators. The layout of the book is very easy to navigate. One of my favorite things as an instructor is that each chapter has clear objectives stated at the beginning of the chapter. While there are not very many pictures in this textbook, each chapter has tables and figures that help explain the content and the topic headings help break things up, so it is easy to read. This is the perfect book for physical educators (adapted or not) to have as a resource for understanding how they can best prepare and teach their students. While this book is focused on adapted physical education many of the topics, strategies and models that are provided could also benefit students without disabilities in physical education.

## Author Contributions

The author confirms being the sole contributor of this work and approved it for publication.

## Conflict of Interest Statement

The author declares that the research was conducted in the absence of any commercial or financial relationships that could be construed as a potential conflict of interest.

